# Assessment and Management of Pain in Patients with Osteoporotic Fragility Fracture

**DOI:** 10.1155/2021/9856174

**Published:** 2021-08-07

**Authors:** Nan Jiang, Wei Ji, Min Yan

**Affiliations:** ^1^Division of Orthopaedics and Traumatology, Department of Orthopaedics, Nanfang Hospital, Southern Medical University, Guangzhou 510515, China; ^2^Division of Spine Surgery, Department of Orthopaedics, Nanfang Hospital, Southern Medical University, Guangzhou 510515, China; ^3^Department of Anesthesiology, The Second Affiliated Hospital of Zhejiang University School of Medicine, Hangzhou 310052, China

Fragility fractures are fractures caused by mechanical forces, known as low-level or low-energy trauma that would not ordinarily lead to fractures. Such forces, quantified by the World Health Organization (WHO), are as forces equivalent to a fall from a standing height or less. As both a sign and a symptom of osteoporosis [1], fragility fractures most frequently occur in the vertebrae, proximal femur, and distal radius [2]. Currently, fragility fractures have become a major public health problem, resulting in high socioeconomic impacts [3–6]. For individuals, fragility fractures often lead to chronic pain, loss of autonomy, deterioration in quality of life, and need for care [5]. Risk factors of fragility fractures include increasing age, postmenopausal females, decreased bone mineral density (BMD), systemic corticosteroid therapy, rheumatoid arthritis (RA), and family history of osteoporosis [2].

Although great advances have been achieved in surgical techniques and instruments for the treatment of fragility fractures, current information of fragility fracture-related pain remains limited. Therefore, we organized this special issue, with the aim of conveying the updated knowledge in the field of evaluation and management of fragility fracture-associated pain.

In this special issue, the readers will find six articles, including three focused on vertebral fractures, two on geriatric hip fractures, and one on foot fractures: “Effect of Preoperative Zoledronic Acid Administration on Pain Intensity after Percutaneous Vertebroplasty for Osteoporotic Vertebral Compression Fractures” by Hu et al.; “Percutaneous Vertebroplasty and Facet Blocking for Treating Back Pain Caused by Osteoporotic Vertebral Compression Fracture” by Cheng et al.; “Advances in Vertebral Augmentation Systems for Osteoporotic Vertebral Compression Fractures” by Long et al.; “Effects of Orem's Self-Care Model on the Life Quality of Elderly Patients with Hip Fractures” by Xu et al.; “Fascia Iliaca Compartment Block for Perioperative Pain Management of Geriatric Patients with Hip Fractures: A Systematic Review of Randomized Controlled Trials” by Wan et al.; and “Prophylaxis of Pain and Fractures within Feet in the Course of Osteoporosis: The Issue of Diagnosing” by Bitenc-Jasiejko et al.

In the field of vertebral fractures, Hu et al. investigated the effects of preoperative administration of zoledronic acid (ZOL) on pain intensity after percutaneous vertebroplasty (PVP) for osteoporotic vertebral compression fractures (OVCFs). Based on a randomized controlled trial (RCT) of 242 patients, the authors concluded that intravenous infusion of ZOL before PVP can effectively decrease the postoperative pain intensity, reduce bone loss, increase bone density, reduce the risk of refracture, and improve the quality of life of the patient. However, the incidence of adverse events in the ZOL group was higher than in the controlled group.

In a retrospective study, Cheng et al. compared the efficacy of PVP plus facet blocking (FB) with PVP alone in relieving postoperative back pain in patients with OVCFs. Based on an analysis of 204 patients, they concluded that PVP combined with FB could provide better pain relief than PVP alone in the short term (1 day and 3 months) after surgery, with similar outcomes at 1-year follow-up time. However, patients receiving the combination method had a longer operation time and more fluoroscopic exposure.

In a review article, Long et al. introduced recent advances on vertebral augmentation systems for the management of OVCFs, including PVP, percutaneous kyphoplasty (PKP), the OsseoFix® system, the SpineJack® system, radiofrequency kyphoplasty (RFK), and the Kiva system. Each technique has advantages and disadvantages, and orthopedists should be familiar with the indications and contraindications of each technique.

Regarding geriatric hip fractures, Xu et al. analyzed the effects of Orem's self-care program on the life quality of senile patients with hip fractures. Based on a RCT of 130 participants, the authors concluded that the self-care program based on Orem's model for geriatric patients with hip fractures could significantly improve life quality and reduce perioperative complications. Whether this nursing program can be routinely applied in this field requires more future studies.

In a systema review, Wan et al. summarized the current evidence of fascia iliaca compartment block (FICB) for perioperative pain management of geriatric patients with hip fractures. Based on comprehensive analyses of 27 RCTs with 2478 cases, the authors concluded that FICB is a safe, reliable, and easy-to-conduct technique, which is able to provide adequate pain relief during perioperative management of geriatric patients with hip fractures. However, as indicated by the authors, due to the still existing flaws of the current RCTs (limited sample size, inconsistency of the outcome parameters, and detailed FICB strategies), future RCTs are warranted.

It is interesting that Bitenc-Jasiejko and colleagues explored the roles of examination of posture and pressure distribution during standing, postural balance, and gait in the prevention of foot fatigue fractures during osteoporosis. Based on the literature review and examples of their clinical patients, they indicated that detailed posture diagnostics and gait estimation, along with the analysis of pressure distribution within the feet, are an essential aspect for the prevention of structural degradation and fatigue fractures within the feet. In addition, they also provided helpful recommendations, which need to be testified in the future.

In this issue, we did not receive submissions on distal radius fragility fractures, which never means the incidence of such a fracture is low. Actually, distal radius fragility fractures possess unique characteristics and treatment [7]. Future studies with a high level of evidence should also focus on this disease.
